# Relationships among Self-Efficacy, Quality of Life, Perceived Vulnerability, and Readiness to Quit Smoking in People Living with HIV

**DOI:** 10.1155/2021/6697404

**Published:** 2021-05-11

**Authors:** Remington E. Donnelly, Haruka Minami, Jacki Hecht, Erika Litvin Bloom, Karen Tashima, Danusha Selva Kumar, Ana Abrantes, Cassandra Stanton, Richard A. Brown

**Affiliations:** ^1^Fordham University, 441 East Fordham Road, Dealy Hall, Bronx, NY 10458, USA; ^2^University of Texas at Austin, 110 Inner Campus Drive, Austin, TX 78705, USA; ^3^RAND Corporation, 20 Park Plaza #920, Boston, MA 02116, USA; ^4^The Miriam Hospital and Alpert Medical School of Brown University, 222 Richmond Street, Providence, RI 02903, USA; ^5^Butler Hospital and Alpert Medical School of Brown University, 345 Blackstone Blvd., Providence, RI 02906, USA; ^6^Westat Inc., 1600 Research Blvd., Rockville, MD 20850, USA

## Abstract

Smoking-related diseases (e.g., lung cancer) are the leading cause of mortality in HIV-infected patients. While many PLWH who smoke report a desire to quit, a majority of them have low readiness to quit. This study used logistic and linear regression to examine the relations among two (continuous vs. binary) measures of readiness to quit, smoking cessation self-efficacy (SE), quality of life (QoL), and perceived vulnerability (PV) using baseline data from 100 PLWH who smoke who participated in a clinical trial. Results showed no significant main effects (SE, QoL, and PV) or interaction effects (SE × QoL and SE × PV) on a continuous measure of readiness to quit. However, a follow-up analysis revealed that SE had a curvilinear effect on readiness to quit such that self-efficacy was positively associated with readiness to quit except at the highest levels of self-efficacy where readiness to quit declined. Greater SE significantly increased the likelihood of reporting readiness to quit (yes/no) among those with low QoL or high PV. For PLWH who smoke, improving self-efficacy may increase readiness to quit especially among those with lower quality of life. Psychoeducation tailored to PLWH designed to reduce unrealistic invulnerability to smoking-related diseases along with interventions that target self-efficacy may improve readiness to quit.

## 1. Introduction

People living with HIV (PLWH) smoke cigarettes at a higher rate than the general population [1–3]. While smoking prevalence declined to around 14.0% in the general population (CDC, 2017), it is estimated that more than 40% of PLWH are smokers [4]. Now that HIV treatment has reduced AIDS-related mortality rates [5, 6], cigarette smoking is a leading cause of morbidity and mortality among PLWH [4, 5, 7, 8]. Compared to nonsmoking PLWH, PLWH who smoke are at increased risk for non-AIDS-defining diseases (e.g., lung cancer, pulmonary disease, and cardiovascular disease) as well as AIDS-defining illnesses (e.g., bacterial pneumonia and tuberculosis) [5, 7, 9–13]. Research has also found that PLWH current smokers have significantly poorer HIV treatment outcomes than former smokers [14]. These findings emphasize the benefits of quitting smoking in improving health outcomes among PLWH.

A significant proportion of PLWH who smoke report a desire to quit, but most do not have an immediate plan to quit smoking [2, 15–17], underscoring that desire to quit does not necessarily translate into readiness or a quit attempt. In fact, Burkhalter et al. [15] reported that over 80% of PLWH who smoke do not have quit plans, and lower readiness is associated with fewer quit attempts. Moreover, readiness and motivation to quit smoking predict an increase in quit attempts [18, 19] as well as cessation success [20]. Therefore, identifying factors that increase readiness may help inform interventions that increase smoking cessation rates among PLWH. The literature suggests that various factors, including self-efficacy, quality of life, and perceived vulnerability (i.e., the extent to which people perceive themselves as being susceptible to negative health impacts of smoking), are related to continued smoking.

In the context of smoking cessation, self-efficacy is often defined as an individual's belief in their ability to quit smoking [21, 22]. Self-efficacy has been extensively studied and shown to be a predictor of motivation to quit and cessation success [21, 23, 24]. Shuter et al. [25] found that higher posttreatment self-efficacy is associated with higher likelihood of cessation success among PLWH. Additionally, a randomized controlled trial found that PLWH who smoke who received a cell phone intervention were more likely to achieve abstinence through an increase in self-efficacy [26]. However, the factors that may moderate the relationship between self-efficacy and quit attempts have not been investigated.

Smoking status in PLWH has also been linked to quality of life measures. Quality of life is influenced by a number of factors including employment, living conditions, social and familial relationships, intimacy, general mood, economic standing, daily functioning, general well-being, overall contentment with life, activities, and physical well-being [27]. Among PLWH, those who smoke report lower overall quality of life than nonsmokers ([28]; Grabovac et al., 2017; [29]), and quality of life is negatively correlated with smoking frequency among PLWH who smoke (Grabovac et al., 2017). Moreover, PLWH who smoke are more likely to be unemployed and have lower income [12, 29] and to report elevated depressive symptoms and less social support [3], compared to nonsmokers. These factors are barriers to cessation; thus, diminished quality of life may reduce readiness to quit.

Perceived vulnerability has also been found to be related to readiness to quit. Perceived vulnerability in the context of smoking cessation has been defined as the extent to which individuals perceive themselves as being susceptible to the negative impacts of smoking [30, 31]. In a smoking cessation study with medically ill patients [30], individuals with higher perceived vulnerability reported greater motivation to quit and increases in perceived vulnerability predicted continued abstinence at the 6-month follow-up period. PLWH have reported low self-efficacy as a barrier to smoking cessation [16]. However, the effects of quality of life or perceived vulnerability on readiness to quit among PLWH have not been well studied. Since quality of life is particularly low among PLWH who smoke [28, 32], it is important to understand these relationships in this population.

The current study examined the relationship among self-efficacy, quality of life, perceived vulnerability, and readiness to quit smoking in PLWH who smoke. Specifically, we hypothesized that (1) higher self-efficacy predicts higher readiness to quit smoking, (2) higher quality of life predicts higher readiness to quit smoking, and (3) higher perceived vulnerability predicts higher readiness to quit smoking. We also explored whether the relationship between self-efficacy and readiness to quit would be moderated by quality of life and perceived vulnerability.

## 2. Method

### 2.1. Participants

This study used baseline data from a completed randomized controlled trial (Brown et al., under review) designed to motivate PLWH to engage in Tobacco Quitline treatment. The original study enrolled 100 smokers, recruited at an outpatient hospital-based HIV clinic in Rhode Island. Inclusion criteria included (1) 18-70 years old, (2) daily cigarette smoker (≥10 cigarettes/day), (3) HIV seropositive, (4) Massachusetts or Rhode Island resident, (5) spoke English, (6) access to a telephone, and (7) available to participate in the study for 3 months. Exclusion criteria included (1) significant cognitive impairment, (2) current use of smoking cessation pharmacotherapy, or (3) use of other tobacco products. The Miriam Hospital Institutional Review Board approved this study, and all participants provided voluntary written informed consent.

### 2.2. Procedure

Research assistants approached participants at the HIV clinic, and individuals who met inclusion criteria were provided with more information about the study. Participants were informed that they did not have to be motivated or ready to quit smoking to participate. All data used for this study were collected via self-report measures and structured interviews at the baseline visit (prior to receiving any interventions).

### 2.3. Measures

#### 2.3.1. Sociodemographic Variables

We collected information on race/ethnicity, income, education level, and relationship status.

#### 2.3.2. Depression

The Center for Epidemiologic Studies-Depression Scale (CES-D) [33] was used to measure depression.

#### 2.3.3. Perceived Stress

The Perceived Stress Scale (PSS) [34] was used to measure stress levels.

#### 2.3.4. Nicotine Dependence

The Fagerström Test for Cigarette Dependence (FTCD) [35] was used as a continuous measure of nicotine dependence.

#### 2.3.5. Readiness to Quit Cigarette Smoking

The Readiness Ruler (RR) was used as a *continuous* measure of readiness to quit cigarette smoking. Participants indicated their readiness to quit cigarette smoking within the next month on a scale of 1 to 10 with 1 = “not at all ready to quit smoking within the next 30 days” and 10 = “actively quitting smoking within the next 30 days” [36]. Participants also answered a *binary* question about whether they were ready to quit smoking in the next 30 days (yes/no).

#### 2.3.6. Smoking Cessation Self-Efficacy

Self-Efficacy (SE) was measured by a 9-item scale assessing participants' confidence in refraining from cigarette smoking in various situations on a 5-point scale with 1 = “Not at all” and 5 = “Extremely” [37].

#### 2.3.7. Quality of Life

The Quality of Life Enjoyment and Satisfaction Questionnaire-Short Form (16 items; Q-LES-Q-SF, [38]) was used to assess physical health, subjective feelings, leisure activities, social relationships, general activities, satisfaction with medications, and life satisfaction domains. The Q-LES-Q-SF has high internal consistency and test-retest reliability [27]. This questionnaire has been shown to be a valid measure of quality of life in a variety of clinical settings, including mood disorders [39] and anxiety disorders [40]. Past studies have used the Q-LES-Q-SF to measure quality of life among PLWH (Jonas et al., 2015; Troeman et al., 2011). Higher scores indicate higher quality of life (QoL).

#### 2.3.8. Perceived Vulnerability

The Perceived Vulnerability Scale consisted of two sections where participants rated the following: (1) the average smoker's risk and (2) their own risk of developing lung cancer, heart disease, and a chronic lung disease other than cancer, on 11 pt scales with 0 = “No Chance” and 100 = “Certain to Happen” (10 pt increments) [41]. Higher scores represent greater perceived vulnerability (PV). For this study, we only used the sum of their ratings of their own perceived risk.

### 2.4. Data Analysis

Frequencies, proportions, means, and standard deviations were used to describe the characteristics of the study sample. We then used the bivariate Pearson correlation, the independent sample *t*-test, and the chi-square test as appropriate to examine the relationship between the characteristics of the study sample and continuous and binary variables of readiness to quit. While only the number of past serious quit attempts was significantly associated with the binary variable of readiness to quit (*r* = 0.20, *p* = 0.044), we included gender, minority status, education level, and nicotine dependence in addition to past quit attempts as covariates in all analyses.

Next, we conducted a series of linear or logistic regression analyses using RStudio, version 1.2.1335. First, a regression analysis was conducted to test the main effects of our predictor variables on the continuous measure of readiness to quit smoking. All three predictors (SE, QoL, and PV) were entered in the same model. We assessed the independent variables and covariates for collinearity. We found that variable inflation factors for all independent variables and covariates were less than 1.61 indicating no or weak correlation between these variables. Next, to examine the moderating effects of QoL and PV on the relation between SE and the continuous measure of readiness to quit, (1) SE × QoL and (2) SE × PV interactions were entered into the main effect model (discussed above) to conduct two additional separate regression models. Second, the same analyses described above were repeated for the binary readiness question using logistic regression. Finally, we conducted an analysis testing the quadratic relationships between all predictors and the continuous measure of readiness to quit smoking. We also ran the analyses using the generalized additive model (GAM), which relaxes assumptions of linearity to capture nonlinear relationships between independent and dependent variables [42]. However, the results remain unchanged; thus, we present our quadratic linear model.

## 3. Results

### 3.1. Participants

Of 100 enrolled participants, 38 (38%) participants were female and 54 (54%) were non-Hispanic White. 63% of the participants reported that they are ready to quit smoking in the next 30 days. The demographic and baseline characteristics for the participants are presented in Table 1.

### 3.2. Continuous Readiness to Quit

#### 3.2.1. Main Effects

A regression model showed that there were no significant main effects of self-efficacy (SE), quality of life (QoL), or perceived vulnerability (PV) on levels of readiness to quit (*p*s > 0.061), controlling for gender, minority status, education, nicotine dependence, and past quit attempts. Separate analyses for each predictor were also conducted, but the significance and direction of the relationships remained unchanged.

#### 3.2.2. Interaction Effects

Two separate regression analyses revealed no significant interaction effects (SE × QoL or SE × PV, *p*s > 0.32) on continuous readiness to quit.

#### 3.2.3. Exploratory Analyses

**Figure 1:**
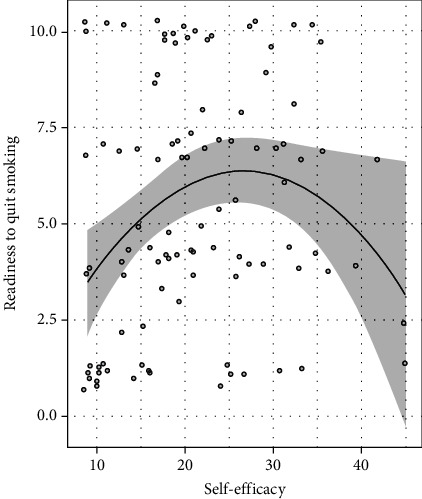
The observed values of self-efficacy and readiness to quit smoking (grey dots: scatterplot) and the predicted values of readiness to quit smoking given the levels of self-efficacy (in black line) and 95% confidence interval (shaded in grey) are depicted. Scatterplot of self-efficacy vs. readiness to quit smoking (continuous) and predicted values of readiness to quit smoking.

A regression model (*R*^2^ = 0.136) showed that SE had significant quadratic (curvilinear) effects on continuous readiness to quit smoking (*β* = −0.010, 95%CI = −0.018, −0.002, *p* = 0.021) (Table 2) such that there is an increase in readiness to quit as self-efficacy increases from low to moderately high (3rd quantile = 27 (of 45)), but readiness to quit declines when self-efficacy becomes higher (Figure 1). However, the plot (depicting scatterplot, predicted outcome, and 95% confidence intervals) also revealed that this curvilinear relationship was driven by several observations with high levels of self-efficacy (≥35, median = 20) with low readiness to quit. No quadratic effects of QoL or PV on readiness to quit were found (*p*s > 0.70) (Table 2).

### 3.3. Binary Readiness to Quit

#### 3.3.1. Main Effects

A logistic regression analysis revealed that neither SE, QoL, nor PV was significantly related to readiness to quit (*p*s > 0.50). The findings remained the same when separate analyses for each predictor were conducted.

#### 3.3.2. Interaction Effects

**Figure 2:**
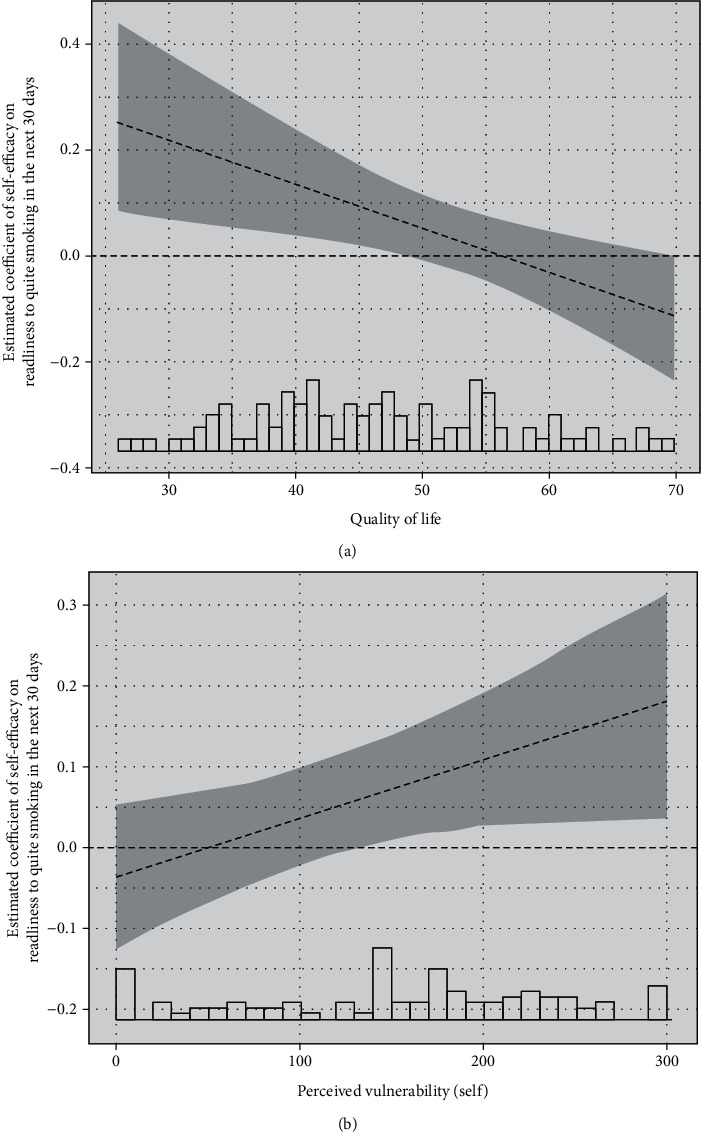
(a, b) The estimated coefficient of self-efficacy on being ready to quit smoking by (a) quality of life and (b) perceived vulnerability is depicted. The estimated coefficient and 95% confidence interval (shaded in grey) were extracted from the logistic regression models. Where the confidence interval does not include zero indicates statistical significance of the coefficient (*p* < 0.05). The histograms of quality of life or perceived vulnerability are shown at the bottom of the corresponding graph. Estimated coefficients of self-efficacy on being ready to quit smoking in the next 30 days.

Two separate regression models showed significant SE × PV (OR = 1.001, 95%CI = 1.000, 1.001, *p* = 0.034) and SE × QoL (OR = 0.992, 95%CI = 0.986, 0.998, *p* = 0.008) interaction effects on being ready to quit smoking (Tables 3(b) and 3(c)). The Hosmer and Lemeshow goodness of fit test for the SE × PV model (*X*^2^ (8) = 7.13, *p* = 0.52) and the SE × QoL model (*X*^2^ (8) = 11.34, *p* = 0.18) indicated no issues with the model fit [43]. Greater SE significantly increased the likelihood of being ready to quit only among those with higher PV and among those with lower QoL. Further analyses revealed that self-efficacy predicted the likelihood of being ready to quit smoking only among those with a quality of life score of 48 or less (57 percentile, median = 47) (Figure 2(a)) and those with a perceived vulnerability score of 135 or greater (30 percentile, median = 175) (Figure 2(b)). When both interaction terms were included in the same model, only SE × QoL remained significant (*p* < 0.05).

## 4. Discussion

The study examined smoking cessation self-efficacy (SE), quality of life (QoL), and perceived vulnerability (PV) in relation to two measures of readiness to quit in the next 30 days (continuous rating scale (from 1 to 10) and binary (yes/no)) in smokers living with HIV. Our hypotheses were partially supported by the binary outcome, but not by the continuous outcome. Contrary to our predictions, neither SE, QoL, nor PV had a significant linear effect on the continuous measure of readiness to quit smoking in the next 30 days, and the relations between SE and continuous measure of readiness to quit did not differ by QoL or PV. However, a follow-up analysis showed that there was a curvilinear relationship between SE and readiness to quit smoking such that while readiness to quit increases with self-efficacy, those with the highest self-efficacy (above 95% percentile) were not likely to report high readiness to quit. The finding that readiness to quit increases with self-efficacy is consistent with previous findings that linked self-efficacy with readiness to quit [44], quit attempts, and cessation success (e.g., [21, 45]; Papadakis et al., 2016). The results from our study adds to these previous findings, as it emphasizes the importance of smoking cessation self-efficacy in predicting readiness to quit in this vulnerable population. The finding that those with highest self-efficacy were not likely to be ready to quit is in line with extant findings that those with very high self-efficacy were less likely to be successful with their cessation [46]. Staring and Breteler [46] found that those in the upper quartile (i.e., 71st percentile) of self-efficacy (i.e., a score of above 20, the possible range = 6‐24) had lower cessation success. They suggest that these participants may be overconfident in their ability to quit smoking, resulting in inadequate use of coping skills. Alternatively, our findings may imply that only those who have not seriously thought about or planned to quit smoking are likely to report extremely high levels of confidence in their ability to quit smoking. In other words, it is possible that extremely high self-efficacy is more likely to reflect low readiness to quit.

However, the relationship between self-efficacy and binary readiness to quit was observed only among those who reported lower QoL and higher PV. Perhaps QoL and PV do not exert much impact on readiness to quit overall, but these factors may set the context for which SE impacts readiness to quit among PLWH. It is possible that for those who experience difficulties in multiple life domains, SE becomes an important determinant of readiness to quit. While this relationship has not been established in smoking cessation studies, it is consistent with research showing that self-efficacy predicts improved self-care behavior among socioeconomically disadvantaged diabetes patients with low quality of life [47]. Similarly, for those with unrealistic beliefs of their invulnerability to smoking-related diseases, SE may be irrelevant in their decision or readiness to quit. However, the SE × PV interaction was no longer significant when the SE × QoL interaction term was included in the model. This suggests that the effect of PV on the relationship between SE and readiness to quit was not beyond what was explained by the moderating effects of QoL on SE. In addition, albeit not a priori hypotheses, the same interaction models can be interpreted in term of the moderating effects of SE. Follow-up analyses on the separate interaction models showed that for those with a SE score of more than or equal to 21 (range 9–45), lower QoL and higher PV predicted higher readiness to quit, respectively, indicating that QoL and PV only predict readiness to quit among those with high SE.

Our findings indicated that SE was not associated with binary readiness to quit but curvilinearly related to continuous readiness to quit. It is possible that the two measures of readiness to quit may have captured different constructs. Closer analysis revealed that these variables were significantly, but only moderately, correlated (*r* = 0.24, *p* = 0.018). Only 54% of those who answered “No” to the binary question (i.e., not ready to quit in the next 30 days) also endorsed “1 = Not at all ready to quit smoking within the next 30 days,” and 37% of them endorsed “4 = Thinking about quitting smoking within the next 30 days” or higher for the continuous question. In addition, among those who answered “Yes” to the binary question (i.e., ready to quit in the next 30 days), 25% of them endorsed “4 = Thinking about quitting smoking within the next 30 days” or less. In other words, the binary question of readiness to quit did not cleanly reflect a dichotomized answer to the continuous measure (e.g., above or below 5). Many participants who endorsed the same score of continuous variable chose a different answer to the binary question. This highlights the importance of how questions are phrased and the differential impact on answers.

The findings from the current study suggest that increasing self-efficacy may help smokers move toward being ready to quit. Several studies have found that the increase in self-efficacy in smoking cessation treatment is linked with higher cessation rates (e.g., [24, 48, 49]), highlighting the important role of self-efficacy in the process of smoking cessation. However, the above studies did not assess whether the increase in self-efficacy is associated with an increase in readiness to quit. Future research is needed to investigate the impact of increasing self-efficacy on readiness to quit among PLWH. Our findings also indicated that self-efficacy may be particularly relevant for PLWH who smoke with lower quality of life and higher perceived vulnerability. Given that many PLWH endorse lower scores in quality of life measures [50–52], it may be especially important to target self-efficacy to help smokers living with HIV reporting multiple life stressors to initiate a quit attempt. The results also underscore the importance of cultivating accurate understanding and awareness around the negative health consequences of smoking, including the specific health concerns for PLWH.

### 4.1. Limitations

There are several limitations of this study. First, the cross sectional and nonexperimental nature of the data preclude our ability to infer causal relations. Future studies examining the effects of changes in the factors on readiness to quit may help elucidate the directions of relationships. Second, our sample size of 100 is underpowered to estimate interaction effects. Additionally, only self-report measures were used in this study. While self-reported readiness to quit has been shown to predict actual quit attempts, actual quit attempts were not assessed. Next, all participants in this study were under 70 years old and recruited from an outpatient hospital-based HIV clinic in Rhode Island, and patients were not consecutively approached. Patients who are receiving treatment at a hospital may have greater health problems, and thus may be more likely to be interested in quitting smoking. Therefore, the findings may not be generalizable to all smokers LWH, especially to those who are not receiving outpatient care for HIV, reside in other regions, or are over the age of 70. In addition, unlike previous findings showing low readiness to quit in a majority of smokers LWH, over 60% of the participants in the current study reported that they were ready to quit smoking in the next 30 days. Although participants were told that they did not have to want to quit smoking in order to participate in the study, it is possible that those who were interested in quitting were more likely to have agreed to participate in this study. At the same time, the sample demographics of this study resemble those in other studies for smokers living with HIV in the Bronx and San Francisco, CA ([53]; Bronx, NY: [25]), although a larger proportion of participants were racial and ethnic minorities in the Shuter et al. [25] study.

## 5. Conclusion

The current study examined the factors related to readiness to quit smoking among smokers LWH. Results suggested that for smokers living with HIV, while extremely high smoking cessation self-efficacy seems to reflect lower readiness to quit, increasing self-efficacy may increase readiness to quit especially among those with poor quality of life and higher perceived vulnerability to smoking-related diseases. Interventions targeting PLWH designed to foster realistic risks of smoking on one's health through psychoeducation and to increase confidence to quit may be helpful in getting smokers living with HIV, especially those with life difficulties, to increase readiness to quit, and thus initiate quit attempts. Future studies investigating the interacting effects of psychosocial factors (e.g., mood, stress, and relationships) associated with readiness to quit as well as actual quit attempts are needed.

## Figures and Tables

**Table 1:** Demographic and baseline characteristics.

	*N* = 100	Ready to quit smoking in the next 30 days (*n* = 63)	Not ready to quit smoking in the next 30 days (*n* = 37)
	*n* (%)		

Female	38 (38%)	26 (41.2%)	12 (32.4%)
Non-Hispanic White	54 (54%)	30 (47.6%)	24 (64.9%)
Latinx	13 (13%)	11 (17.4%)	2 (5.4%)
Education			
AA or higher	16 (16%)	9 (14.3%)	7 (18.9%)
Some college	20 (20%)	12 (19.0%)	8 (21.6%)
HS diploma	19 (19%)	13 (20.6%)	6 (16.2%)
Some HS	31 (31%)	19 (30.2%)	12 (32.4%)
Less than HS	13 (13%)	10 (15.9%)	3 (8.1%)
Household income			
$100,000 or more	3 (3%)	3 (4.8%)	0 (0.0%)
$75,000-$99,999	2 (2%)	0 (0.0%)	2 (5.4%)
$50,000-$74,999	6 (6%)	2 (3.2%)	4 (10.8%)
$25,000-$49,999	8 (8%)	4 (6.3%)	4 (10.8%)
$0-24,999	80 (80%)	54 (85.7%)	26 (70.3%)

	Mean (SD)		

Age	48.80 (9.03)	49.27 (7.83)	48.00 (10.84)
Cigarettes per day	17.05 (8.20)	16.77 (7.93)	17.23 (9.52)
Nicotine dependence (FTCD)	5.63 (2.17)	5.68 (2.23)	5.54 (2.09)
Smoking cessation self-efficacy (range 9-45)	21.07 (8.48)	22.46 (7.84)	18.70 (9.10)
Quality of life (range 14-70)	47.41 (10.14)	47.00 (9.77)	48.14 (10.85)
Perceived vulnerability (range 0-300)	160.2 (83.92)	165.08 (81.32)	151.89 (88.69)

*Note.* FTCD = Fagerström Test for Cigarette Dependence.

**Table 2:** Quadratic effects of perceived vulnerability, self-efficacy, and quality of life on a continuous measure of readiness to quit smoking.

	*β*	95% CI	*p*
Intercept	-1.759	(-15.203, 11.685)	0.799
Past quit attempts	0.184	(-0.076, 0.444)	0.170
Gender	-0.014	(-1.436, 1.409)	0.985
Non-Hispanic White	-0.529	(-1.922, 0.863)	0.458
Education	-0.394	(-1.775, 0.986)	0.577
FTCD	0.124	(-0.191, 0.438)	0.443
Perceived vulnerability (linear effect)	0.002	(-0.031, 0.027)	0.899
Perceived vulnerability^1^ (quadratic effect)	0.000	(-0.000, 0.000)	0.701
Self-efficacy (linear effect)	0.510	(0.132, 0.889)	0.010^∗^
Self-efficacy^1^ (quadratic effect)	-0.010	(-0.018, -0.002)	0.021^∗^
Quality of life (linear effect)	0.045	(-0.492, 0.583)	0.870
Quality of life^1^ (quadratic effect)	-0.001	(-0.006, 0.005)	0.796

*Note*. ^1^ = squared term. FTCD = Fagerström Test for Cigarette Dependence.

**Table:** (a) Main effects of perceived vulnerability, self-efficacy, and quality of life on a binary measure of readiness to quit smoking

	OR	95% CI	*p*
Intercept	0.176	(0.003, 10.700)	0.407
Past quit attempts	1.242	(0.992, 1.555)	0.059
Gender	1.806	(0.639, 5.108)	0.265
Non-Hispanic White	0.410	(0.156, 1.074)	0.070
Education	0.701	(0.263, 1.868)	0.477
FTCD	1.115	(0.887, 1.401)	0.351
Perceived vulnerability	1.005	(0.998, 1.012)	0.161
Self-efficacy	1.057	(0.995, 1.124)	0.073
Quality of life	0.984	(0.935, 1.034)	0.519

*Note.* FTCD = Fagerström Test for Cigarette Dependence.

**Table:** (b) Self − efficacy × perceived vulnerability interaction effect on a binary measure of readiness to quit smoking

	OR	95% CI	*p*
Intercept	0.437	(0.013, 14.793)	0.645
Past quit attempts	1.239	(0.995, 1.544)	0.056
Gender	1.531	(0.515, 4.550)	0.443
Non-Hispanic White	0.387	(0.143, 1.049)	0.062
Education	0.594	(0.212, 1.663)	0.321
FTCD	1.163	(0.914, 1.480)	0.219
Perceived vulnerability	0.993	(0.979, 1.006)	0.289
Self-efficacy	0.969	(0.879, 1.068)	0.529
Self − efficacy × perceived vulnerability	1.001	(1.000, 1.001)	0.034^∗^

*Note.* FTCD = Fagerström Test for Cigarette Dependence.

**Table:** (c) Self − efficacy × quality of life interaction effect on a binary measure of readiness to quit smoking

	OR	95% CI	*p*
Intercept	0.001	(0.000, 0.472)	0.029^∗^
Past quit attempts	1.326	(1.050, 1.675)	0.018^∗^
Gender	1.044	(0.366, 2.972)	0.936
Non-Hispanic White	0.405	(0.150, 1.095)	0.075
Education	0.633	(0.227, 1.766)	0.382
FTCD	1.099	(0.869, 1.391)	0.431
Self-efficacy	1.585	(1.152, 2.181)	0.005^∗∗^
Quality of life	1.135	(1.002, 1.285)	0.046^∗^
Self − efficacy × quality of life	0.992	(0.986, 0.998)	0.008^∗^

*Note.* FTCD = Fagerström Test for Cigarette Dependence.

## Data Availability

The data used to support the findings of this study are available from Dr. Richard Brown (brown2@utexas.edu) upon request.
